# Microbes from Mum: symbiont transmission in the tropical reef sponge *Ianthella basta*

**DOI:** 10.1038/s43705-022-00173-w

**Published:** 2022-09-27

**Authors:** J. Pamela Engelberts, Muhammad A. Abdul Wahab, Manuel Maldonado, Laura Rix, Emma Marangon, Steven J. Robbins, Michael Wagner, Nicole S. Webster

**Affiliations:** 1 Australian Centre for Ecogenomics, School of Chemistry and Molecular Biosciences, The University of Queensland, Brisbane, QLD Australia; 2 Australian Institute of Marine Science, Townsville, QLD Australia; 3 Department of Marine Ecology, Centre for Advanced Studies of Blanes (CEAB-CSIC), Girona, Spain; 4 College of Science and Engineering, James Cook University, Townsville, QLD Australia; 5 Centre for Microbiology and Environmental Systems Science, Department of Microbiology and Ecosystem Science, University of Vienna, Vienna, Austria; 6 Center for Microbial Communities, Department of Chemistry and Bioscience, Aalborg University, Aalborg, Denmark; 7 Australian Antarctic Division, Kingston, TAS Australia

**Keywords:** Microbial ecology, DNA sequencing, Marine microbiology

## Abstract

Most marine sponge species harbour distinct communities of microorganisms which contribute to various aspects of their host’s health and physiology. In addition to their key roles in nutrient transformations and chemical defence, these symbiotic microbes can shape sponge phenotype by mediating important developmental stages and influencing the environmental tolerance of the host. However, the characterisation of each microbial taxon throughout a sponge’s life cycle remains challenging, with several sponge species hosting up to 3000 distinct microbial species. *Ianthella basta*, an abundant broadcast spawning species in the Indo-Pacific, is an emerging model for sponge symbiosis research as it harbours only three dominant symbionts: a Thaumarchaeotum, a Gammaproteobacterium, and an Alphaproteobacterium. Here, we successfully spawned *Ianthella basta*, characterised its mode of reproduction, and used 16S rRNA gene amplicon sequencing, fluorescence in situ hybridisation, and transmission electron microscopy to characterise the microbial community throughout its life cycle. We confirmed *I. basta* as being gonochoric and showed that the three dominant symbionts, which together make up >90% of the microbiome according to 16S rRNA gene abundance, are vertically transmitted from mother to offspring by a unique method involving encapsulation in the peri-oocytic space, suggesting an obligate relationship between these microbes and their host.

## Introduction

Sponges are found in marine and freshwater environments from the tropics to the poles, playing a particularly important role in the survival and productivity of coral reefs where they recycle and retain (in)organic nutrients [1, 2]. Their evolutionary success is, in part, derived from the metabolic integration between the sponge host and its diverse community of symbiotic microbes [3, 4]. For example, sponge-associated microbes can remove waste products, provide the host with nutrients, and produce secondary metabolites that aid in host defence [5–9]. The host must ensure high fidelity of transmission of important microorganisms [10], therefore these sponge symbionts are expected to be heritable, either through vertical transmission (from the parent), horizontal transmission (from the environment), or a combination of both (i.e. ‘mixed mode’; [11–16]).

Symbiont acquisition mode can shape sponge phenotype, particularly when microbes mediate important developmental stages. For example, vertically inherited symbionts of the sponge *Amphimedon queenslandica* supply their host with the amino acid arginine, something the host cannot produce itself [17–19], and arginine is also used to produce nitric oxide, a signalling compound that triggers settlement and metamorphosis of *A. queenslandica* larvae [20]. Vertical transmission ensures the faithful transfer of microbes critical to host survival. However, sponges that rely solely on vertical transmission can lose the ability to adapt to changing environments [21, 22]. Horizontal transmission, where microbes are taken up from the surrounding seawater, allows for the acquisition of genetically diverse microbial strains that may enable the host to better acclimatise to changing environmental conditions [12, 23, 24]. However, this mode of transmission can lead to the loss of beneficial symbionts as well as to the acquisition of pathogens [11, 21, 25]. The mode and mechanism of transmission can thus provide insight into the nature of the host-symbiont relationship, yet for most sponge species the mechanisms of symbiont transmission remain unknown. The development of model systems where the microbiome is fully characterised throughout the host life cycle would greatly facilitate studies on how individual symbionts mediate host development and adaptation [17].

*Ianthella basta* is an oviparous, verongiid demosponge, a group for which little is known about their sexual reproduction [26, 27]. This sponge harbours only three dominant symbionts that make up >90% of the microbial community: a Thaumarchaeotum, a Gammaproteobacterium and an Alphaproteobacterium [7, 28]. The remaining <10% of the microbial community is composed of low abundant or transitory taxa. This reduced microbial complexity enables the characterisation of the mode of transmission for each symbiont throughout the sponge’s life cycle, a task that is intractable in sponges with more complex microbiomes. Here, we successfully spawned *I. basta* in indoor aquaria, confirmed its mode of reproduction, and characterised its late-stage gametes. Using a combination of 16S rRNA gene amplicon sequencing, fluorescence in situ hybridisation (FISH) and transmission electron microscopy (TEM) we characterised the microbial community of *I. basta* adults and offspring. We reveal that all dominant symbionts are vertically transmitted through a unique mechanism, where microbes are packaged into the peri-oocytic space before the release of the oocyte into the seawater, ensuring fidelity of transmission, and incorporation into the oocyte after its release.

## Materials and methods

### Sponge collection and broodstock maintenance

*Ianthella basta* presents in two colour morphotypes, purple and yellow [29]. To ensure consistency, seven purple adult *I. basta*, with a relatively thick morphology, were collected on the 6th of August 2018 around the predicted peak reproduction period, when water temperature rises above 23.4 °C [27, 30], from Cattle Bay, Orpheus Island (S 18°35.259', E 146°28.883*'*) using SCUBA at depths between 9 and 12 m. Sponges were immediately transferred to indoor flow-through aquaria in the National Sea Simulator at the Australian Institute of Marine Science (AIMS) in Townsville, Australia. Three female individuals containing oocytes, as determined via visual inspection of gametes in the sponge mesohyl, were moved into two 884 L rectangular aquaria tanks (930 mm width × 1440 mm length × 760 mm height; water height = 660 mm), and a pair of putative male sponges (those not containing oocytes) were placed in each tank to allow for fertilisation upon egg release. Tank 1 contained two female sponges (Ib126 and Ib134) and two potential male sponges (Ib127 and Ib132) and Tank 2 contained one female sponge (Ib130) and two potential male sponges (Ib128 and Ib131).

Broodstock holding tanks were supplied with flow-through 0.4 μm filtered seawater (FSW; flow rate = 2 L min^−1^, ~3 × 100% water exchange per 24 h) in a temperature-controlled room maintained at 25 °C. Unfiltered seawater (RSW, 1 L min^-1^) was supplemented to each tank daily for ~6 h during the day to provide sponges natural particulate and dissolved matter as nutrition. The temperature of in-flow water supply to the aquaria was adjusted according to the 2010 daily average seawater temperature recorded at Orpheus Island at 10 m depth, ranging between 23.1 °C to 26.4 °C from the 1st of August to the 30th of September. Seawater temperature was controlled by mixing two streams of temperature-controlled FSW using automatic valves regulated through a Siemens PCS7 SCADA system. Water circulation in each tank was maintained using 1 × Panta Rhei Hydrowizard (Flow A: 20% [0.6 s], Flow B: 1% [0.6 s], Interval flow A–B = 0.1 s).

Photosynthetically active radiation (PAR) was supplied to the aquaria using LED aquarium lights (Hydra, AquaIllumination), reproducing natural irradiance and lighting regimes experienced by sponges in situ (https://researchdata.edu.au/orpheus-island-light-sep-2017). The lighting regime comprised a linear ramp-up period of 6.5 h from darkness (0530 h) to a maximum of 5 μmol quanta m^−2^ s^−1^ (1200 h) and a ramp-down over 6.5 h to darkness (1830 h). A dim white LED was additionally installed to simulate the moon phases throughout the experiment. This LED light was set to 1 Lux, equivalent to the outdoor full moon intensity, and was proportionally dimmed to the new moon to replicate natural moonlight overnight.

### Spawning and sample collection

To detect spawning and collect gametes (i.e. oocytes and sperm) released by sponges and/or embryos after external fertilisation, the overflow discharge from tanks was drained into a 75 L cylindrical gamete catcher with a conical bottom. A meshed central standpipe, with a gentle air curtain, allowed for gametes to be concentrated within each tank without damaging or losing gametes through the drainage. Gamete catcher tanks were checked three times daily for gametes at 0800, 1200 and 1600 h. Upon spawning, oocytes and embryos were concentrated and collected from the catcher tanks by gently washing with 0.4 μm filtered seawater (FSW) and draining through 495, 150 and 28 μm mesh sieves to remove any large foreign particles. Fertilisation occurred in Tank 1, but not in Tank 2 (see ‘Results’). Consequently, embryos were sampled from Tank 1 on the first (*T* = 1, *n* = 3) and second day (*T* = 2, *n* = 3) of spawning, covering the full duration of the spawning event. From Tank 2, unfertilised eggs were collected on the second day of spawning (*T* = 2, *n* = 3). Each sample contained at least 20 embryos (Tank 1) or oocytes (Tank 2). Embryos were also collected for larval rearing and developmental observations (see Supplementary note 1). Furthermore, tissue from all adult *I. basta* was sampled during spawning (*T* = 1, *n* = 7 and *T* = 2, *n* = 7), as well as one day post spawning (*T* = 3, *n* = 7; total *n* = 21). Seawater was collected from both tanks (*n* = 2 per tank), as well as from the water inflow into the tanks, which alternated between raw seawater (RSW, *n* = 1) and 0.4 μm filtered seawater (FSW, *n* = 3; see ‘Sponge collection and broodstock maintenance’). Upon collection, one litre of seawater was filtered through 0.2 µm Sterivex filters (Millipore) and filters were snap frozen and stored at −80 °C until DNA extraction and 16S rRNA gene amplicon sequencing.

All *I. basta* tissue samples (i.e. oocytes, embryos, and adults) were preserved for DNA extraction and 16S rRNA gene amplicon sequencing, fluorescence in situ hybridisation (FISH), and transmission electron microscopy (TEM, Table S1). Samples were rinsed in 0.2 µm filtered seawater, snap frozen (oocytes/embryos) or preserved in absolute ethanol (adults), and stored at −80 °C until DNA extraction. For FISH, samples were fixed in 4% paraformaldehyde (PFA) for 6–24 h at 4 °C and stored in 1:1 ethanol‐phosphate‐buffered saline (PBS) at −20 °C. For TEM, samples were fixed in a buffer consisting of 25% glutaraldehyde, 20% PFA, and 0.4 µm-filtered seawater and stored at 4 °C. Samples were rinsed and re-fixed in 2.5% glutaraldehyde in 0.2 M Millonig’s phosphate (MP) buffer (pH 7.4) and 0.14 M NaCl for 1 h, rinsed again in MP buffer for 40 min, and post-fixed in 2% OsO_4_ in 0.2 M PB for 1 h. Samples were subsequently rinsed in Milli-Q water, dehydrated in a graded series of ethanol, infused with propylene oxide, and embedded in Spur resin. For histological processing (haematoxylin and eosin staining), tissue samples from all adult sponges were fixed in FAACC (100 mL = 10 mL of 40% formaldehyde, 5 mL of glacial acetic acid, 1.3 g of calcium chloride dehydrate and 85 mL of tap water; [31] on the day after field collection, and at *T* = 1, 2 and 3 (*n* = 7 per time point).

### DNA extraction, 16S rRNA gene amplicon sequencing, and sequence analysis

DNA was extracted from *I. basta* adult tissue and seawater using the DNeasy Powersoil Kit (Qiagen, Germany), following the manufacturer’s instructions, including a bead beating step of 40 s at 2500 rpm. Due to the fragile nature of the oocytes and embryos, DNA was extracted using a standard SDS/Proteinase K lysis method [32]. Extracted DNA was sent to the Australian Centre for Ecogenomics (ACE, Brisbane) for sequencing of the V4 region of the 16S rRNA gene, using the primers 515F [33] and 806R [34] targeting both bacteria and archaea and having no mismatches to the 16S rRNA genes of the three major symbionts of *I. basta*. 16S rRNA gene libraries were prepared using the workflow outlined by Illumina (#15044223 Rev.B) followed by sequencing on an Illumina MiSeq Sequencing system (2 × 300 bp).

Raw sequences were quality filtered and trimmed with Trimmomatic [35], keeping sequences with an average base quality above 15 and minimum length of 250 bp. Quality controlled reads were processed using QIIME2 v2020.11.1 [36] for feature selection, abundance calculations, and taxonomic assignment. Chimeric sequences were removed with DADA2 [37]. Taxonomy was assigned to features, referred to as amplicon sequence variants (ASVs) from here on, by BLASTing its sequence against the SILVA database (release 138, clustered at 99% identity; http://www.arb-silva.de/). Chloroplasts, mitochondrial sequences, and singletons were removed from the dataset. For Alpha diversity calculations, the ASV table was rarefied to an even sequencing depth of 2300 reads. Beta diversity calculations were performed on the unrarefied ASV table.

Statistical analyses were performed in R v3.5.1 [38]. Statistical differences between Alpha diversities were assessed with analyses of variance (ANOVAs). Bray Curtis dissimilarities were calculated for all samples and visualised in a two-dimensional space by Non-metric Multi-dimensional Scaling (NMDS, ‘phyloseq package’). To determine similarity between the microbiomes of *I. basta* adults and offspring and to test whether the microbial community of *I. basta* was significantly different from the surrounding seawater, analyses of similarity (ANOSIM, ‘vegan package’) and permutational multivariate analyses of variance (PERMANOVAs as implemented in the adonis2 function, ‘vegan package’, 999 permutations) were performed, respectively. Multivariate Homogeneity of Groups Dispersions (betadisper, ‘vegan package’) were subsequently analysed to account for potential differences in group variance. To calculate and display the percentage of the microbial community shared between *I. basta* life stages, a weighted Venn diagram was created using the function ‘ps_venn’ (https://github.com/Russel88/MicEco/tree/v0.9.15). An ASV was counted as present in a life stage when identified in >60% of the samples. In addition, a differential abundance analysis was performed using Aldex2 v1.24.0 [39] to identify individual ASVs that differed significantly in their relative abundance across life stages (test = ‘Wilcox’). Graphs were created using ggplot2 v3.3.5 and refined in Inkscape v0.92.4 (https://inkscape.org/).

### Cryosectioning and fluorescence in situ hybridisation (FISH)

Samples preserved for FISH (i.e. tissue of *I. basta* adults, oocytes, and embryos) were washed for 1 h in 1× PBS and stored overnight at 4 °C in 30% sucrose to preserve tissue morphology. Samples were transferred to 50% sucrose in equal volume with embedding medium (1:1, Surgipath FSC 22 Frozen Section Embedding Medium) and stored overnight at 4 °C. Samples were subsequently frozen in the embedding medium and sectioned to 5 μm on a Thermofisher Cryostar NX70 cryostat. Sections were transferred onto gelatin-coated microscope slides and stored at −20 °C until further processing.

For FISH, slides were dehydrated for 3 min each in 50%, 80%, and 96% (v/v) ethanol and dried at 46 °C. Hybridisation buffer (10 µl; 5 M NaCl, 1 M Tris/HCl, 30% formamide, 10% sodium dodecyl sulfate (SDS) was added to each sample with 1 μl of either the EUB338 I–III probe mix [40], ARCH915 probe [41] complementary to the abundant thaumarchaeal symbiont *Candidatus* Nitrosospongia ianthellae [7], or recently designed probes specific to the dominant Alpha- and Gammaproteobacterial symbionts of *I. basta* [42], termed AlfD729 and GamD1137, respectively (double-labelled [43], final probe concentration ~2.5 ng/μl). As negative controls, sections were hybridised with a double-labelled NON338 [44] or without a probe (autofluorescence). Slides were hybridised for 3 h at 46 °C and incubated in pre-heated (48 °C for 3 h) washing buffer (5 M NaCl, 1 M Tris/Hcl, 0.5 M EDTA) for 15 min at 48 °C. Slides were dipped twice for 2–3 s into ice-cold double distilled water (ddH_2_O), immediately air dried, mounted with DABCO antifade mounting medium, and visualised on a ZEISS LSM 510 META laser scanning confocal microscope.

### Transmission electron microscopy (TEM)

Ultrathin sections of Spur blocks were obtained with an Ultracut Reichert-Jung ultramicrotome, mounted on gold grids, and stained with 2% uranyl acetate for 30 min and lead citrate for 10 min. Samples were processed at the Microscopy Services of the University of Barcelona on a JEOL 1010 transmission electron microscope operating at 80 kV and provided with a Gatan module for digital imaging. TEM images were taken of two male and two female sponges sampled on the day of spawning, as well as of recently released unfertilised oocytes.

To confirm that the microbial morphotypes identified in the spawning *I. basta* individuals were consistently found across other *I. basta* individuals, three more adult specimens were collected in October 2020 and processed for TEM imaging at the Australian Centre for Ecogenomics (ACE), Brisbane (see Supplementary note 2).

## Results

### Spawning, mode of reproduction, and gamete characteristics

*Ianthella basta* spawning was first detected on the 12th of August 2018 at 0815 h, one day after the new moon (11th of August 2018 at 1957 h). Histological sections were assessed only after spawning had occurred, consequently Tank 1 (with two female sponges) contained two sexually mature male sponges whereby fertilisation successfully occurred, while Tank 2 (with one female sponge) contained two male individuals that were sexually immature. Therefore, embryos and larvae were collected from Tank 1, and unfertilised eggs were collected from Tank 2. Sperm and oocytes never co-occurred in the same individual sponge, confirming *I. basta* as gonochoric.

**Fig. 1:**
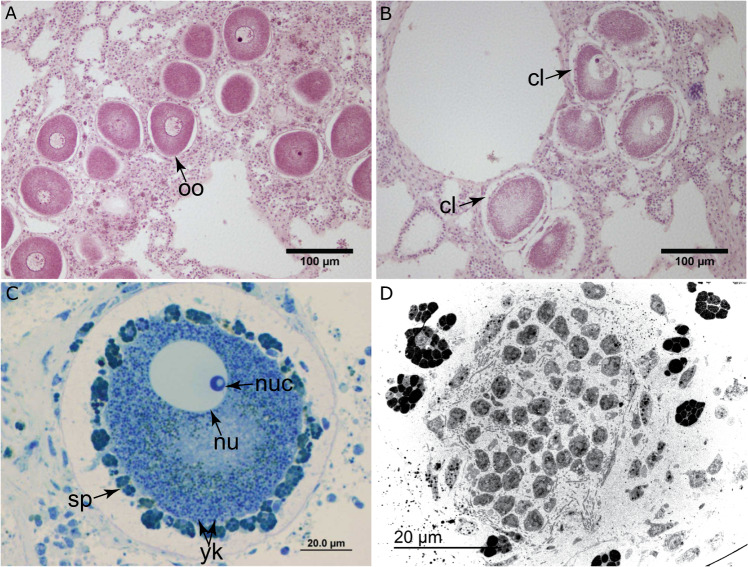
Micro-photographs and microscopy images of developing oocytes and a spermatyc cyst. Micro-photographs of **A** female *I. basta* 5 days before spawning, showing oocytes (oo) without a sheath of maternal cells, **B** the same female *I. basta* on the day of spawning showing mature oocytes surrounded by a sheath of maternal cell layer (cl), and **C** a late-stage oocyte showing the nucleolus (nuc), nucleus (nu), yolk bodies (yk), and spherulous cells (sp). **D** Transmission electron microscopy image of a lobe of a spermatic cyst in tissue from an *I. basta* male sampled during the first day of spawning.

Histological preparations revealed that spermatogenesis lasted only a few days, with spermatic cysts detected in samples on the day of spawning, but not in samples collected five days prior. Spermatic cysts were localised near the aquiferous canals and were 164.56 ± 39.13 μm long and 42.78 ± 6.83 μm wide (mean ± SE, *n* = 5), with the mean diameter of individual spermatic cells measuring 2.76 ± 0.04 μm (*n* = 30, Fig. S1A). TEM images of male tissue on the day of spawning showed spermatic cysts at an immature stage, containing mostly secondary spermatocytes (Figs. 1D and S1B–D). Consequently, spermiogenesis (i.e. the final stage of spermatogenesis) and maturation to produce functional sperm must occur rapidly (minutes to hours) to contribute to a synchronic male gamete release event. Alternatively, remaining immature sperm may contribute to a secondary pulse of sperm release as eggs were observed in females on the third day after the main spawning event. Microbial cells were absent from both the cytoplasm of all investigated secondary spermatocytes and the lumen of the spermatic cysts (Fig. S1).

**Fig. 2:**
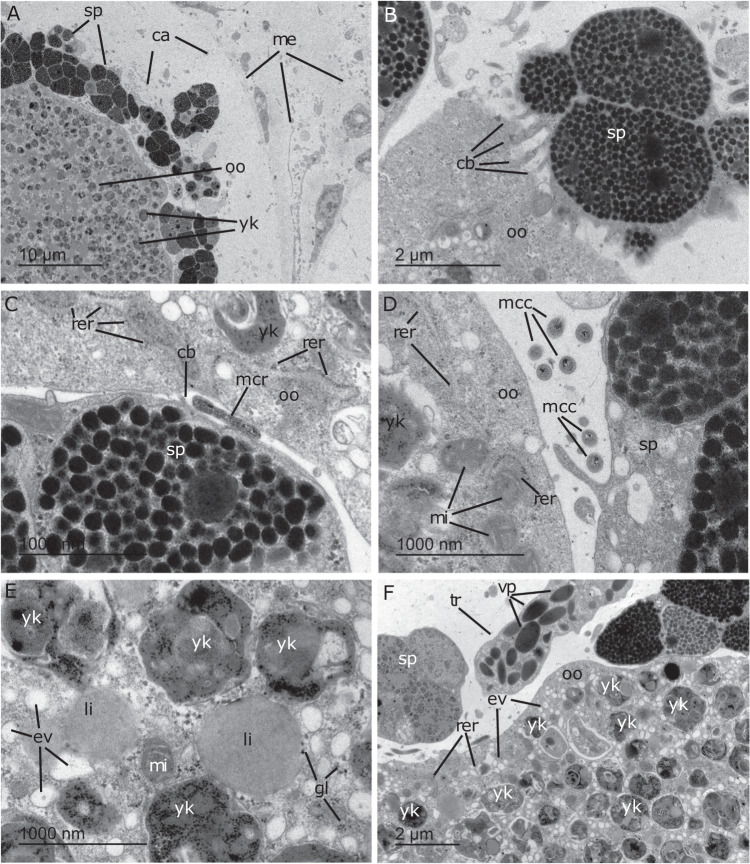
Transmission electron microscopy images of an unreleased egg in *Ianthella basta*. **A** Partial view of a late-stage oocytes (oo) in female tissue sampled during the first day of spawning. Oocytes are still hosted in a cavity (ca) within the mesohyl (me) and surrounded by a layer of maternal spherulous cells (sp). **B** The spherulous cells (sp) may establish cytoplasmic bridges (cb) for the transfer of maternal nutrients to the oocyte (oo). **C**, **D** Detail of the space between the spherulous cell and the oocyte, named peri-oocytic space, in which rod-like microbes (mcr, diameter: 0.17–0.28 µm), as well as small, coccoid microbes (mcc, diameter: 0.10-0.18 µm) occur. Microbes were absent from the cytoplasm of the unreleased eggs. **E** The oocyte cytoplasm at an inner region, showing the absence of symbionts and an abundance of complex yolk bodies (yk), lipid droplets (li), glycogen granules (gl), mitochondria (mi), and electron clear vesicles (ev). **F** An oocyte in which a trophocyte (tr), a maternal cell charged with vitelline platelets (vp), has been incorporated into the follicle of spherulous cells (sp). The peripheral cytoplasm of the oocyte (oo) contains numerous yolk bodies (yk), rugose endoplasmic reticulum (rer), and electron clear vesicles (ev).

Mature oocytes had a maximum diameter of 87.93 ± 1.60 μm (*n* = 56) within adult sponges which increased slightly when released (Tank 2; diameter: 98.98 ± 0.62 μm, *n* = 100; Figs. 1B and S2B). During their late phase in the mesohyl, oocytes became surrounded by an epithelium-like envelop of maternal cells, which reorganised into a follicle (Figs. 1A–C, 2A, and S3A). This sheathing of oocytes was detected within 5 days leading up to spawning (Fig. 1A, B) and gave oocytes the dark red coloration of adult sponges (Fig. S2A, B). The follicle consisted of two maternal cell types: spherulous cells and trophocytes. Spherulous cells are large membrane-bound spherules that contained numerous membrane-bound microgranules (Figs. 2A–C and S3B). Spherulous cells were the dominant cell type in the follicle and were occasionally seen embraced by pseudopodia emitted from the oocyte (Figs. 2A, B and S3A, B). Furthermore, cytoplasmic bridging occurred between the spherulous cells and oocyte (Fig. 2B, C), suggesting the transfer of content to the growing oocyte. The follicle also occasionally incorporated trophocytes, cells charged with vitelline platelets or other yolk reserves that could be transferred to the maturing oocyte (Fig. 2F). The innermost region of the oocyte cytoplasm was packed with large bodies of complex yolk, lipid droplets, glycogen granules, mitochondria, and electron clear vesicles (Fig. 2E). In addition to these organelles, the peripheral cytoplasm contained rugose endoplasmic reticulum with well visible ribosomes and mitochondria (Figs. 2F and S3F). These features indicate that prior to their release, oocytes are heavily involved in the production of messenger RNA and proteins.

**Fig. 3:**
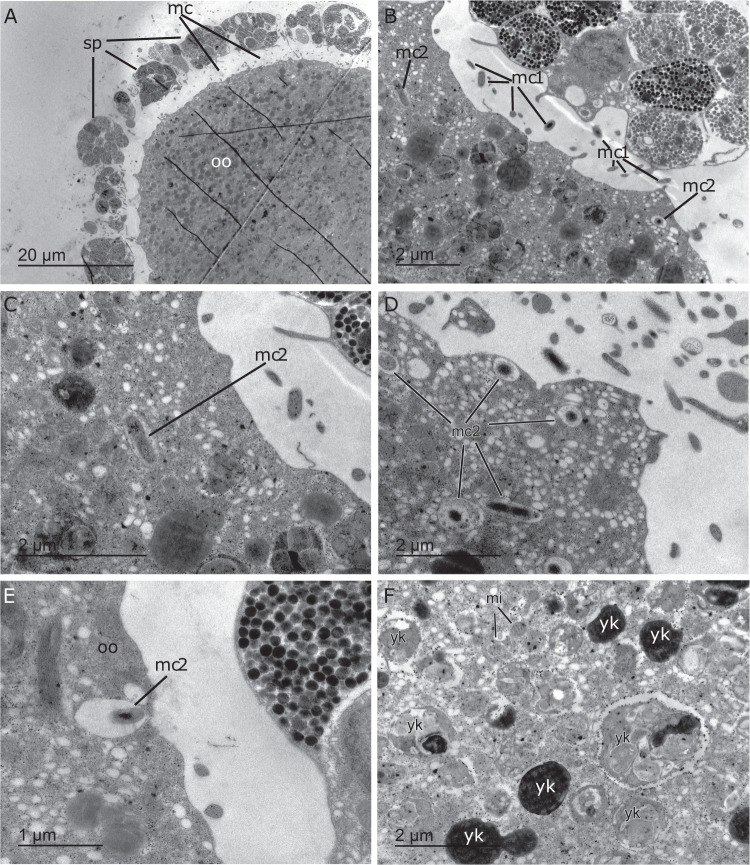
Transmission electron microscopy images of a released, unfertilised egg of *Ianthella basta*. **A** Partial view of a spawned oocyte (oo), which is expelled along with its follicle of spherulous maternal cells (sp) and microbial cells (mc) that were hosted in the peri-oocytic space. The cracks in the resin through the cytoplasm of the oocyte were caused by a defective embedding in the Spur resin. **B** Periphery of the spawned egg in which some of the microbial symbionts (mc1) still remain in the peri-oocytic space and some (mc2) have already started being incorporated into the peripheral cytoplasm. **C**, **D** Microbial cells (mc2) incorporated by the egg after spawning, some of which are dividing. **E** Microbial cell (mc2) recently phagocytosed by the oocyte (oo) from the peri-oocytic space. **F** Inner region of the cytoplasm of the released egg, which shows the typical organelles, such as yolk bodies (yk) and mitochondria (mi), and the absence of symbiotic microbes, which are currently only distributed throughout the peripheral cytoplasm.

Spawning was noticed in the tanks over 2 days, with approximately 60% of oocytes released on the first day of spawning and 81% of all oocytes released by the second day of spawning. The oocytes remaining (~20%) in the mesohyl of females, as observed on the third day of spawning, were not surrounded by a layer of maternal cells. The fate of these immature oocytes, i.e. whether they were eventually released or resorbed, could not be determined. Upon release, eggs were negatively buoyant and still surrounded by the maternal envelope, which consisted mostly of spherulous cells (Fig. 3A). In contrast, the unsheathing of the maternal cell layer was commonly observed within the first 2 days of gamete release in unfertilised oocytes of *I. basta* (Fig. S2B).

### Larval characterisation, settlement, and juvenile development

At ~24 h post-fertilisation, larvae were 113.48 ± 1.58 μm long and 83.03 ± 1.04 μm wide (Tank 1, *n* = 54, Fig. S2C). After 3 d, larvae attached to the substrate within a minute of introduction to petri dishes, flattening their body structures to resemble discs. Visible aquiferous systems were detected at 12-days post-settlement (Fig. S4), indicating that individual sponges were able to pump, and likely filter, water at this early stage of their life history.

### Microbial diversity of *I. basta* adults, offspring, and seawater

To characterise *I. basta*’s microbial community and elucidate the mode of symbiont transmission, DNA was extracted and sequenced from *I. basta* male and female adults, offspring (i.e. individual oocytes as well as embryos) and seawater (tank, filtered inflow = FSW, and unfiltered inflow = RSW). In total, 2,872,908 raw sequence reads were obtained, of which 1,250,296 remained after filtering. In the filtered reads, a total of 5149 amplicon sequence variants (ASVs) were identified based on single nucleotide variations, which were classified into 7 archaeal and 56 bacterial phyla (Table S2). Seawater samples had the highest richness (with on average 297 ± 37 ASVs for FSW and RSW and 256 ± 47 ASVs for seawater in the tanks; Table 1). Richness was lowest in *I. basta* adults (22 ± 14 ASVs), followed by oocytes and embryos (48 ± 17 ASVs and 62 ± 48 ASVs, respectively). Shannon indices (i.e. alpha diversities) were significantly different between the seawater and *I. basta* adults and offspring (ANOVA: *F*_(4/33)_ = 108.6, *p* < 2e-16), but not between *I. basta* life stages (TukeyHSD: *p* > 0.05).

**Table 1:** Richness, Shannon index, and evenness (mean and standard deviation; sd) for *I. basta* adults (*n* = 7 per time point: *n* = 4 males, *n* = 3 females, see also Fig. 4B) and offspring (*n* = 9: *n* = 3 oocytes, *n* = 6 embryos), seawater in the tanks (SW_Tanks, *n* = 4), and seawater inflow into the tanks (SW_Inflow, *n* = 4), which alternated between raw seawater (RSW, *n* = 1) and filtered seawater (FSW, *n* = 3).

Group	Richness (mean)	Richness (sd)	Shannon (mean)	Shannon (sd)	Evenness (mean)	Evenness (sd)
Adults	21.62	13.63	1.52	0.28	0.52	0.08
Females	26.89	19.55	1.59	0.31	0.52	0.08
Males	17.67	4.5	1.47	0.25	0.52	0.08
Offspring	57.44	39.11	1.6	0.54	0.42	0.1
Oocytes	48.33	16.74	1.31	0.1	0.34	0.02
Embryos	62	47.54	1.75	0.62	0.47	0.1
SW_Tanks	256.25	46.96	4.22	0.32	0.76	0.03
SW_Inflow	296.75	37.37	4.5	0.14	0.79	0.03
RSW	259	NA	4.53	NA	0.82	NA
FSW	309.32	33.83	4.49	0.16	0.78	0.04

### Broad-scale differences in the microbial communities of *I. basta* adults, offspring, and seawater

**Fig. 4:**
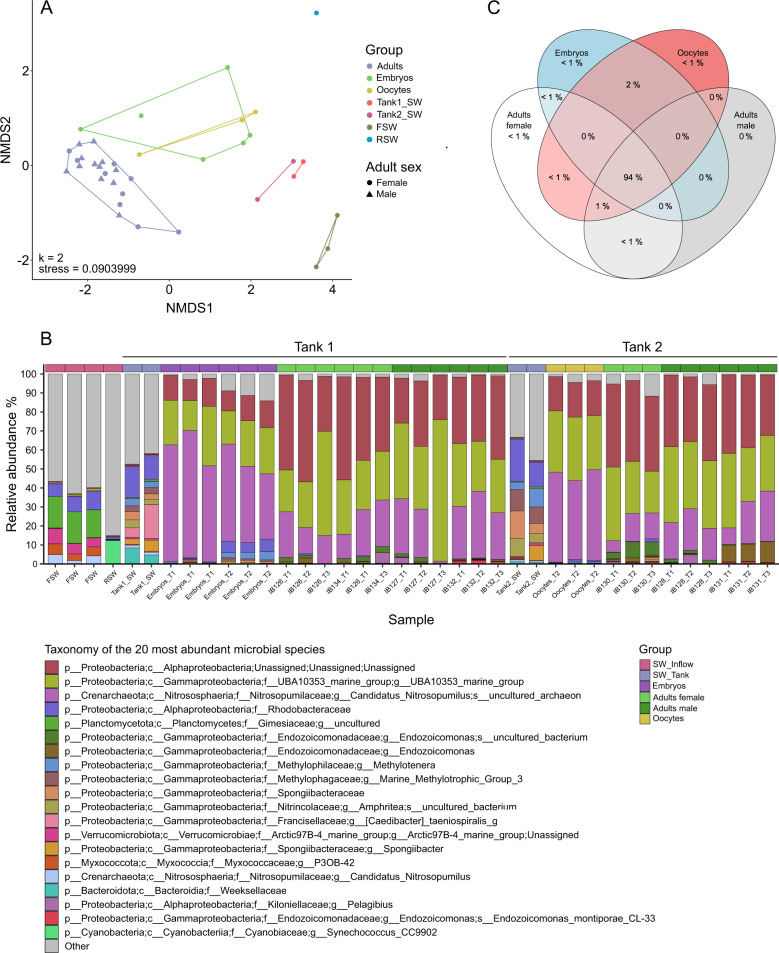
Microbiome similarity, composition, and overlap throughout *I. basta*’s life stages. **A** Non-metric multidimensional scaling plot displaying the (dis)similarity in the microbial community of *I. basta* adults, oocytes, and embryos, seawater from Tank 1 and 2 (Tank1_SW and Tank2_SW, respectively), and seawater inflow into the tanks, which alternated between filtered (FSW) and raw seawater (i.e. unfiltered; RSW). **B** Barplot displaying the 20 most abundant microbial species in *I. basta* adults (with females displayed in light green and males in dark green), oocytes, embryos, seawater in the tanks (SW_Tank), and seawater inflow into the tanks (SW_Inflow). The lowest resolved taxonomy is shown, for simplicity displaying phylum, class, family, genus, and species. FSW: Filtered seawater, RSW: Raw seawater (i.e. unfiltered), Ib: *Ianthella basta*, followed by individual number and timepoint sampled, with T1 and T2 being the first and second day of spawning and T3 the day after spawning. **C** Weighted Venn diagram displaying the percentage of the microbial community shared between groups. ASVs were present in a group when found in >60% of the samples, which not differed significantly in sequencing depth. The list of unique and shared ASVs can be found in Table S4.

In the absence of a tank effect on microbiome composition (Adonis2: *p* > 0.05), samples from Tank 1 and 2 were pooled together per group for beta diversity calculations (see Table 1 for groups). The microbial communities of *I. basta* adults and offspring were significantly different from those found in the surrounding tank seawater and seawater inflow (Adonis2: *p* < 0.01; Fig. 4A). The microbiome composition of adult males and females also varied significantly (Adonis2: *p* < 0.01), and both sexes were kept separate in further analyses. Further, the microbiomes of adult males and females were significantly different from the microbiomes of oocytes and embryos (Adonis2: *p* < 0.01; ANOSIM (adults vs offspring): *p* = 0.001, *R* = 0.83), while oocytes and embryos themselves did not vary significantly in their microbial communities (Adonis2: *p* > 0.05; ANOSIM: *p* = 0.372, *R* = 0.031).

### A Thaumarchaeotum, Gammaproteobacterium, and Alphaproteobacterium dominate *I. basta*’s microbiome throughout all life stages

Although the microbiome of *I. basta* adults and offspring varied significantly, it was always dominated by three ASVs: a Thaumarchaeotum (*Candidatus* Nitrosospongia ianthellae, ASV_5060), Gammaproteobacterium (genus-level: UBA10353_marine_group, ASV_803), and Alphaproteobacterium (unclassified, ASV_2141; Fig. 4B). Adults had lowest relative abundance of the Thaumarchaeotum (females: 14 ± 6%, males: 20 ± 9%, Fig. S5A), followed by the Gammaproteobacterium (females: 30 ± 11%, males: 37 ± 13%, Fig. S5B), and Alphaproteobacterium (females: 41 ± 8%, males: 31 ± 6%, Fig. S5C). A reverse pattern was seen in oocytes and embryos, where the Thaumarchaeotum dominated the microbiome (oocytes: 46 ± 3%, embryos: 48 ± 11%), followed by the Gammaproteobacterium (oocytes: 31 ± 2%, embryos: 23 ± 5%), and Alphaproteobacterium (oocytes: 18 ± 0%, embryos: 13 ± 2%). All three ASVs were absent from the seawater inflow (Figs. 4B and S5, Table S2), with the exception of 19 Thaumarchaeotum reads recovered from one of the filtered seawater samples. Less than 0.1% of the seawater microbial community in the tanks containing the sponges consisted of the three dominant ASVs (Figs. 4B and S5). Notably, ASV_5060 showed 100% sequence identity to the publicly available genome of *I. basta*’s Thaumarchaeota symbiont *Candidatus* Nitrosospongia ianthellae [7], ASV_803 showed 99.60% sequence identity to the genome of *I. basta*’s gammaproteobacterial symbiont from the LS-SOB clade [42], and ASV_2141 showed 99.20% sequence identity to the genome of *I. basta*’s Alphaproteobacterial symbiont from the order JABSOH01.

The software Aldex2 was used to identify ASVs that were significantly different in their relative abundance between *I. basta* life stages and sexes. Of the three dominant ASVs, only the Thaumarchaeotum had a significantly lower relative abundance in male adults than offspring (Aldex2: p < 0.05). A further seven ASVs were significantly different between male adults and offspring and one between female adults and offspring (Table S3). ASV_3522 (class Gammaproteobacteria, genus Methylotenera) showed the highest increase in relative abundance in offspring compared to adults (Aldex2: *p* < 0.01), while ASV_596 (class Gammaproteobacteria, genus Endozoicomas) showed the highest decrease in relative abundance in offspring, in which it was absent, compared to male adults (Aldex2: *p* < 0.01). Adult males and females did not differ significantly in the relative abundance of individual ASVs, neither did oocytes and embryos (Aldex2: *p* > 0.05).

### Shared and unique ASVs throughout *I. basta*’s lifecycle

To visualise shared and unique ASVs in the microbial communities of *I. basta* adults and offspring, a weighted Venn diagram was generated showing that 94% of the microbial community was shared between adults and offspring (Fig. 4C, Table S4). This primarily comprised the three dominant symbionts (ASV_5060, ASV_803, and ASV_2141) and ASV_2137 (class Gammaproteobacteria, genus Marine_Methylotrophic_Group_3; Table S3). However, it must be noted that ASV_2137 had an average relative abundance of <0.4% in *I. basta* samples, while having an average relative abundance >6% in the surrounding seawater. Unique to female and male adults was ASV_4387 (class Gammaproteobacteria, genus Endozoicomonas, Table S4). This ASV was absent from offspring and the surrounding seawater. In contrast, ASV_5291 and ASV_596 (class Gammaproteobacteria, genus Endozoicomonas) were uniquely shared between adults and oocytes and were further present in half of the embryo samples and absent from the seawater. Female adults shared ASV_4786 (class Chlamydiae, order Chlamydiales) with oocytes. Although this ASV was absent from the seawater inflow, it was only present at a very low abundance in *I. basta* (0.13% in female adults and 0.08% in oocytes). No ASVs were solely shared between male adults and oocytes and/or embryos.

### Presence of dominant symbionts in *I. basta* adults and offspring supported by FISH

As *Ianthella basta* is an oviparous sponge, adults contain (unfertilised) gametes in their tissue prior to spawning (Fig. 1). Fluorescence in situ hybridisation (FISH) was used to visualise microbial symbionts around and/or in these gametes to further elucidate the mode and mechanisms of symbiont transmission. Here, we focused on the visualisation of the three dominant symbionts in late-stage oocytes in female adults as well as in eggs and embryos collected from seawater.

**Fig. 5:**
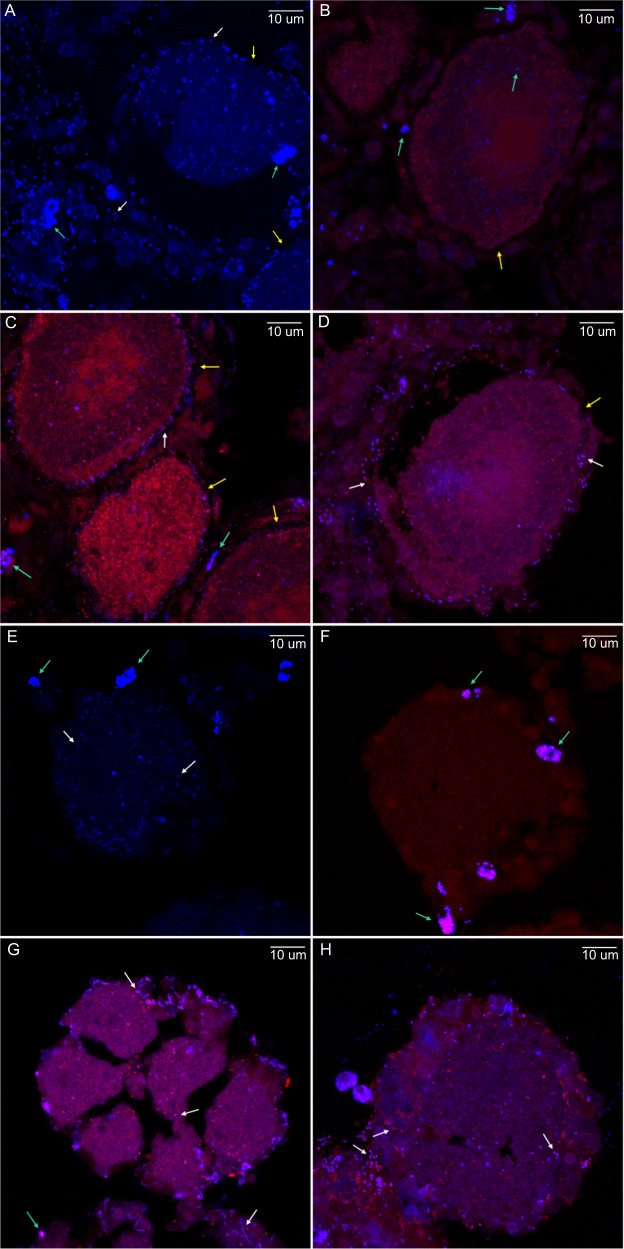
Fluorescence in situ hybridisation (FISH) of *Ianthella basta* adults and offspring. **A**–**D** Female adult tissue containing oocytes not yet released into the surrounding seawater and **E**–**H** *I. basta* offspring (oocytes and embryos) collected from the aquarium seawater. Yellow arrows point towards the oocytes, green arrows towards non-specific binding of probes, and white arrows towards bacteria (red) or one of the three dominant symbionts (blue). Tissue was hybridised with **A** and **E** the general ARCH915 probe in Cy5 (blue), **C** and **G** the general EUBI-III probe in Cy3 (red), and the Gammaproteobacterium specific probe in Cy5 (blue), **D** and **H** the general EUBI-III probe in Cy3 (red) and the Alphaproteobacterium specific probe in Cy5 (blue). **B** and **F** Signals observed with control probe NON338 in Cy3 (red) and Cy5 (blue).

*I. basta* tissue from adults containing late-stage oocytes was hybridised with FISH probes targeting most bacteria (EUB I-III in Cy3) and archaea (i.e. targeting *Candidatus* Nitrosospongia ianthellae, the only numerically relevant Thaumarchaeotum present in *I. basta*, ARCH915 in Cy5). Bacteria and archaea were present in the adult tissue, as well as directly around the oocytes (Fig. 5A, C, D), although some non-specific probe binding occurred (see also the NON338 in Cy3 and Cy5; Fig. 5B). Bacteria were absent from oocytes, while the Thaumarchaeotum was occasionally observed in oocytes (Fig. 5A). Hybridisation of the adult tissue with EUB I-III in Cy3 and symbiont specific probes (i.e. probes specific to the dominant Gamma- and Alphaproteobacterium in Cy5) showed that these two microbes directly surrounded the oocytes in the female adult tissue (Fig. 5C and D, respectively). Notably, the Gammaproteobacterium had a rod-shaped morphology, with an approximate length of 1 µm and width of 0.1 µm, while the Alphaproteobacterium and Thaumarchaeotum resembled coccoid cells, with an approximate diameter of 0.5 µm (Fig. 5A, C, D, E, G, and H).

Oocytes and embryos collected from the seawater after spawning were also hybridised with the probe targeting most archaea (ARCH915 in Cy5), revealing the presence of *Candidatus* Nitrosospongia ianthellae in *I. basta*’s offspring (Fig. 5E). Non-specific probe binding only occurred outside the oocyte, with similar signals found in the negative control (NON338 in Cy3 and Cy5; Fig. 5F). Hybridisation of oocytes and embryos with EUB I-III in Cy3 and symbiont specific probes in Cy5 revealed that the Gammaproteobacterium was located around the oocytes/embryos and occasionally within the oocytes/embryos (Fig. 5G). The dominant Alphaproteobacterium was localised both around and in the oocytes/embryos (Fig. 5H).

### TEM supports the vertical transmission of microbes

TEM images used to describe gamete characteristics (i.e. Figs. 2, 3 and S3) were also analysed for the presence of microbes. In the mother adult tissue, the space between the layer of spherulous cells and the oocyte was found to be occupied by prokaryotes of two morphologies (Figs. 2C, D and S3C–F, see also Figure S6): 1) small, coccoid cells (<200 nm in diameter) and 2) larger, rod-like cells (>500 nm in length). The latter could correspond to the Gammaproteobacterial symbiont, as a similar size and morphology were seen in the FISH images. Based on morphology and abundance, the coccoid cells could correspond to the Alphaproteobacterium and/or Thaumarchaeotum. TEM screening of the cytoplasm of late-stage oocytes also showed that prokaryotic cells, either free in the intracellular medium or within vesicles, were absent (Figs. 2C–F and S3B, F).

Once eggs were expelled into the water column, they preserved their surrounding populations of prokaryotic symbionts, hosted in the space between the layer of maternal spherulous cells and the oocyte itself (Fig. 3A, B). Microbes were subsequently incorporated by the oocyte into its peripheral cytoplasm (Fig. 3C, E). During incorporation, microbial cells became enclosed into individual vesicles and did not show signs of digestion, instead began proliferating via division (Fig. 3C, D). The acquired microorganisms were predominantly concentrated in the peripheral cytoplasm, with the inner regions of the egg being dominated by yolk bodies and other organelles (Fig. 3F).

Notably, the microbial morphologies identified in the released eggs were similar to the microbial morphologies found in the adult tissue of *Ianthella basta*. These morphotypes were also seen in the tissue of three *I. basta* adults sampled in September 2020 (Fig. S6) as well as a previous study on healthy and diseased *I. basta* [28], confirming that these morphotypes are consistently found over time and across *I. basta* individuals.

## Discussion

Marine sponges harbour distinct communities of microbes, that can shape sponge phenotype by mediating important developmental stages and influencing the environmental tolerance of the host. However, the characterisation of each microbial taxa throughout a sponge’s life cycle remains challenging. Here, we characterised the microbiome of the model sponge *Ianthella basta* throughout its life cycle to facilitate future studies on how individual symbionts mediate host development and adaptation. To this end, we successfully spawned the oviparous sponge *I. basta*, confirming it is a gonochoric species (separate male/female sexes) that releases gametes after the new moon in August on the Great Barrier Reef. We subsequently characterised the microbiome throughout all life stages of the sponge and revealed that the three dominant sponge symbionts are vertically transmitted from female adults to oocytes through a unique mechanism. In this mechanism, microbes are packaged into the space between spherulous cells and the oocyte (termed peri-oocytic space) before the release of the oocyte into the seawater and are incorporated into the oocyte after its release, suggesting an obligate relationship between these symbionts and their host.

To characterise *I. basta*’s life cycle at the start of development, we first investigated gamete production to determine the mode of gametogenesis. Reproductively mature *I. basta* individuals simultaneously released gametes in aquaria 1-day after the new moon in August 2018, consistent with previous field observations in which *I. basta* contained gametes in August 2010, but not in September 2010 [27]. Spermatogenesis in *I. basta* occurred within 5-days of broadcast spawning and male and female gametes did not co-occur in individuals, confirming *I. basta* is gonochoric. This finding follows the general pattern of gonochorism being the dominant sexual phenotype for oviparous sponges, with 13 out of the 22 orders belonging to the Demospongia showing gonochorism [45]. The microbial community of *I. basta* was subsequently characterised throughout the sponge’s life cycle using 16S rRNA gene amplicon sequencing. The microbiome of both adults and offspring was dominated by three symbionts (i.e. a Thaumarchaeotum, Gammaproteobacterium, and Alphaproteobacterium) and was significantly different from the surrounding seawater. The three dominant sponge symbionts were present in low abundance in all tank water (<0.1% abundance) and absent from the inflowing seawater, with the exception of an extremely low abundance of the Thaumarchaeotum in one of the four seawater replicates. As these Thaumarchaeotum reads likely result from a contamination during sequencing, we suggest that the observed signals of all three dominant symbionts in the tank water originated from the sponge host and conclude that the symbionts are vertically transferred. Vertical transmission of the three dominant symbionts would also be expected as the larvae and embryos are lecithotrophic (reliant on internal yolk reserves for nourishment). Horizontal acquisition of microbes generally occurs post-larval settlement and metamorphosis when the aquiferous system develops [46–49], and recruits are actively filter feeding [23, 50], which occurred in *I. basta* juveniles between 5 and 12 days post-settlement.

To determine the mechanism of vertical transmission of the three dominant symbionts, we further processed oocytes in the mother adult tissue as well as released oocytes and embryos for FISH and TEM. In other commonly studied reef species, like broadcast spawning corals or the sea anemone *Nematostella vectensis*, microbes are thought to be transmitted through the mucus surrounding the gametes, while brooding corals could seed bacteria into developing larvae [51–54]. In sponges, symbionts are most commonly transferred prior to release of the eggs or larvae via both phagocytosis and other mechanisms. For example, in the viviparous homosclerophorid *Corticium candelabrum*, symbionts aggregate around late-stage oocytes, which are initially engulfed and digested as food [55] and then migrate into the intercellular spaces of the embryo during cleavage after the egg is internally fertilised [12]. In other species, symbionts are initially phagocytosed from the mesohyl by nurse cells which subsequently transfer the symbionts to the oocytes through cytoplasmic bridges e.g. [50]. In contrast, in *I. basta*, FISH indicated that the three dominant symbionts aggregate directly around the oocytes in the adult tissue (Fig. 5), without being incorporated, which was supported by TEM (Fig. 2). Although FISH images showed that the Thaumarchaeotum occasionally occurred within the oocytes, this was not supported by TEM observations despite quantitative FISH analysis indicating that archaea should be present at sufficient numbers to be detectable by TEM (see Supplementary note 3 and Table S5). The same discrepancy between FISH and TEM images (i.e. a signal versus no detection) has previously been reported in oocytes from the oviparous sponge *Ectyoplasia ferox* [50] and may be an artefact of sample processing for FISH (i.e. fixation, embedding, and sectioning for FISH might transfer some cells). Taken together, the results suggest that the three dominant symbionts surround oocytes, but do not directly inhabit the oocyte while inside the mother tissue. However, upon release into the seawater, both FISH and TEM showed the incorporation of microbes, which included the three dominant symbionts. Interestingly, TEM showed that symbionts were expelled along with the egg, hosted in the peri-oocytic space between the oocyte and follicle membrane of maternal cells, and incorporated into the egg through phagocytosis before the disintegration of the envelope of maternal cells (Fig. 3). Thus, we show that symbiont incorporation takes place only after the eggs are released into the water column. This timing differs from the method of symbiont incorporation of the only two other species in the order Verongiida studied to date, *Aplysina cavernicola* and *Aplysina aerophoba*, which phagocytose microbial symbionts from the mesohyl into late-stage oocytes prior to rather than post release [26, 56]. The novel mechanism of vertical transmission identified in *I. basta* ensures fidelity of transmission, maintains a tight association between *I. basta* and its microbial community, and suggests an obligate relationship between the dominant symbionts and their host.

Once transmitted to new offspring, the dominant Thaumarchaeotum occurred at a significantly higher relative abundance (20 ± 9% in adults, 46 ± 3% in oocytes and 48 ± 11% in embryos), potentially indicating its importance in *I. basta*’s development. One way through which *Candidatus* Nitrosospongia ianthellae could mediate developmental stages is by producing nitric oxide through nitrite reduction catalysed by its highly expressed nitrite reductase NirK [7]. Nitric oxide is a signalling compound that induces metamorphosis in the sponge *A. queenslandica* [20, 57]. It also regulates metamorphosis in various other marine invertebrates [58–61], where it either induces or inhibits settlement. Whether the Thaumarchaeotum and the compound nitric oxide are involved in the settlement and metamorphosis of *I. basta* remains to be confirmed, thus further experiments to clarify the role of the Thaumarchaeotum and nitric oxide in sponge larval settlement and metamorphosis are required.

While all three dominant symbionts are vertically transferred, our data also suggest that several other microbial taxa may be acquired post-settlement, likely when the sponge begins pumping. Although present in low abundance, these microbes can be repositories of key functions [62]. Hence, we also characterised their potential mode of transmission. ASV_4786, belonging to the order Chlamydiales, of which the members are obligate intracellular symbionts, was found in female adults and oocytes and is likely acquired vertically by oocytes. In contrast, ASV_4387 (class Gammaproteobaceria, genus Endozoicomonas) is putatively horizontally transmitted (i.e. taken up from the seawater when the sponge starts pumping) as it was only present in adults and seawater.

Here we identified the mode of transmission of the major microbiome members in the tropical reef sponge *I. basta*. We show that the three dominant symbionts, comprising >90% of the microbial community according to 16S rRNA gene abundance, are vertically transmitted through a unique mechanism. This mechanism involves the encapsulation of microbes in the peri-oocytic space and the incorporation of the microbes into the oocytes after their release into the surrounding seawater, suggesting an obligate relationship between these microbes and their host. Consequently, this study provides a valuable framework for future manipulative experiments to study the role of the three dominant symbionts in the development of *I. basta*. Furthermore, it established a fully characterised model organism to further study the molecular mechanisms for symbiont acquisition in demosponges.

## Supplementary information

Supplementary Information

Table S1

Table S2

Table S3

Table S4

Table S5

## Data Availability

All sequencing data from this study are available under BioProject ID PRJNA779745 from the NCBI Sequence Read Archives.
