# Supplementary material for: Microbes from Mum: symbiont transmission in the tropical reef sponge Ianthella basta
Source: ISME Commun. 2022 Sep 27;2:90. doi: 10.1038/s43705-022-00173-w (PMC9723589; doi:10.1038/s43705-022-00173-w)
Supplement: Supplementary file 1 — Supplementary Information [file 43705_2022_173_MOESM1_ESM.pdf]

Supplementary Information

Microbes From Mum: Symbiont transmission in the tropical reef sponge *Ianthella basta*

J. Pamela Engelberts, Muhammad A. Abdul Wahab, Manuel Maldonado, Laura Rix, Emma Marangon, Steven J. Robbins, Michael Wagner, Nicole S. Webster

Materials and methods

Supplementary note 1

Embryos from Tank 1 were transferred into individual 20 L conical culture tanks with gentle aeration. Larvae in culture tanks were harvested after three days and allowed to settle and metamorphose onto clean polystyrene petri dishes (100 × 20 mm) containing FSW on a bench in the experimental room over a period of 24 h. Petri dishes with settlers were subsequently transferred back into the broodstock holding tanks for grow-out and developmental observations.

Supplementary note 2

Tissue from three *I. basta* specimens collected in September 2020 from Davies Reef (S 18°49.354’, E 147°38.253’) was dissected into small pieces (~1 mm^3) and immediately fixed in 3% glutaraldehyde and 1% paraformaldehyde in buffer containing 0.2M sodium cacodylate, 0.35M sucrose, and 0.1M sodium chloride for 4 h at room temperature followed by overnight fixation at 4 °C. Samples were then transferred to buffer (0.2M sodium cacodylate, 0.3M sodium chloride) and stored at 4 °C until further processing. All subsequent steps were performed in a Pelco Biowave (Ted Pella, Ca, USA). Samples were post-fixed in 1% osmium tetroxide in sodium cacodylate, stained with 1% uranyl acetate, dehydrated in ethanol (30%, 50%, 70%, 90%, and 2X100%), and embedded in EPON resin before polymerization at 60°C for 48 h. Ultrathin sections (~80 nm) were cut using a diamond knife and an Ultracut UC6 microtome (Leica Microsystems, Australia) and stained with uranyl acetate and lead

citrate prior to imaging on a Hitachi HT7700 Transmission Electron Microscope at the Centre for Microscopy and Microanalysis (University of Queensland).

Supplementary note 3

FISH images of oocytes still residing in the mother tissue were analysed quantitatively with ImageJ (1) to confirm the presence of archaea and reveal whether archaea could have been missed in TEM sections due to a low abundance. To this end, archaea were counted in oocytes that were imaged at 1000x magnification and numbers were corrected by subtracting counts detected after hybridization with the control probe NON338. Counts were further normalized to um^3 oocyte (assuming an ellipse shape of the oocyte), as well as to a 5 µm FISH section and a 100 nm TEM section. Up to 270 archaea were found per oocyte if analyzed by FISH per 5 µm thick sections. Thus, a 100 nm TEM section of an oocyte should contain up to 5.4 archaea, but no microbes were observed (**Table S5**).

Supplementary Table description

Table S1. Overview of the type of samples collected during this experiment, the time of collection, and for what analyses the samples were collected. FISH: Fluorescence *in situ* hybridization. TEM: Transmission electron microscopy.

Table S2. Counts, abundances, and taxonomy of identified amplicon sequence variants across samples.

Table S3. Aldex2 output showing the significantly different amplicon sequence variants between adult females and offspring and adult males and offspring.

Table S4. Shared and unique ASVs across *Ianthella basta* life stages.

Table S5. Quantitative FISH calculations of *Candidatus* Nitrosospongia ianthellae in oocytes for images captured at 1000x magnification.

Supplementary Figures

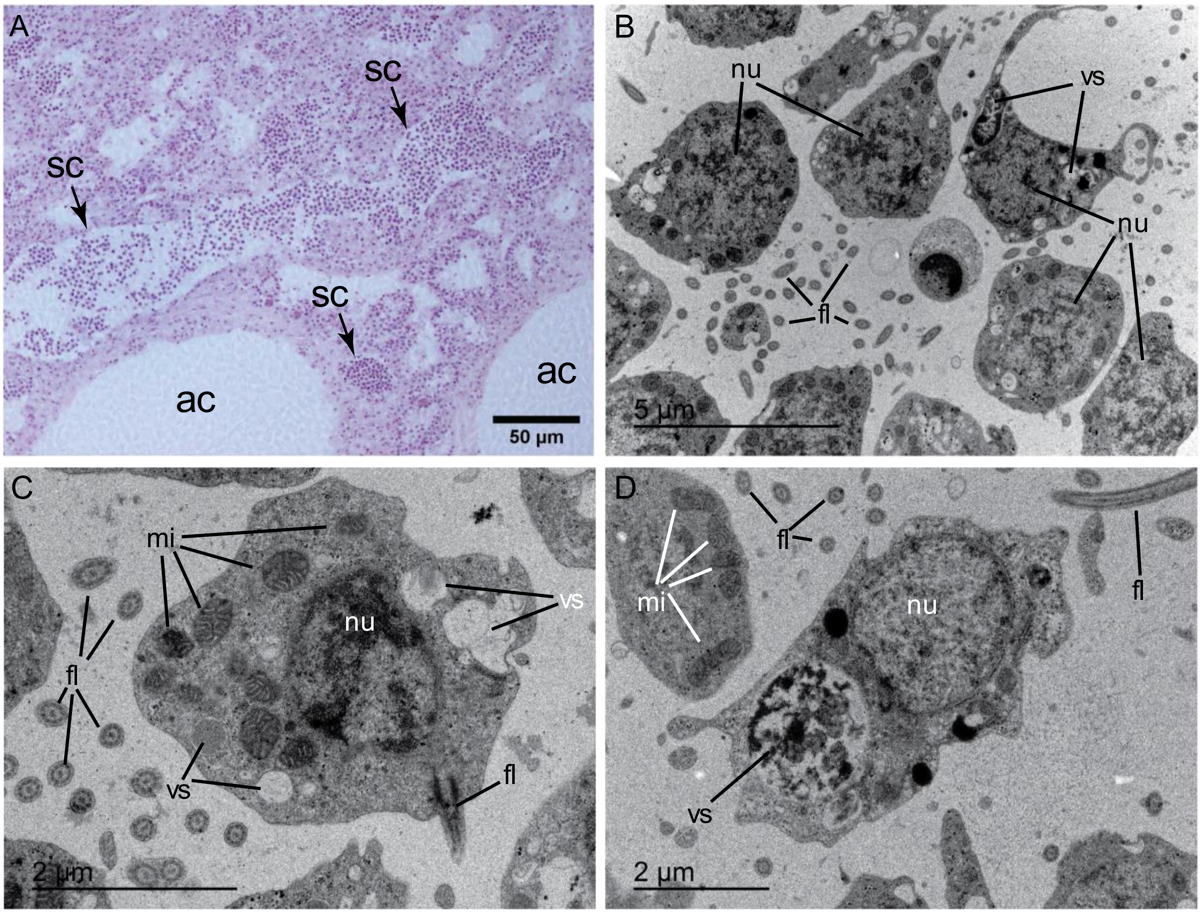

Figure S1. A) Micro-photographs of male *I. basta* exhibiting spermatic cysts (sc) that are localized adjacent to aquiferous canals (ac). B-D) Transmission electron microscopy images of a spermatic cyst in *Ianthella basta*. Within the cyst, the spermatogenesis is still at the stage of secondary spermatocyte, as indicated by large nuclei (nu) with non-condensed chromatin, a cytoplasm still containing diverse vesicles with inclusions (vs), and small mitochondria (mi) that have not fused yet. Microbial cells did not occur in the lumen of the cyst or within the spermatocytes. fl: flagella.

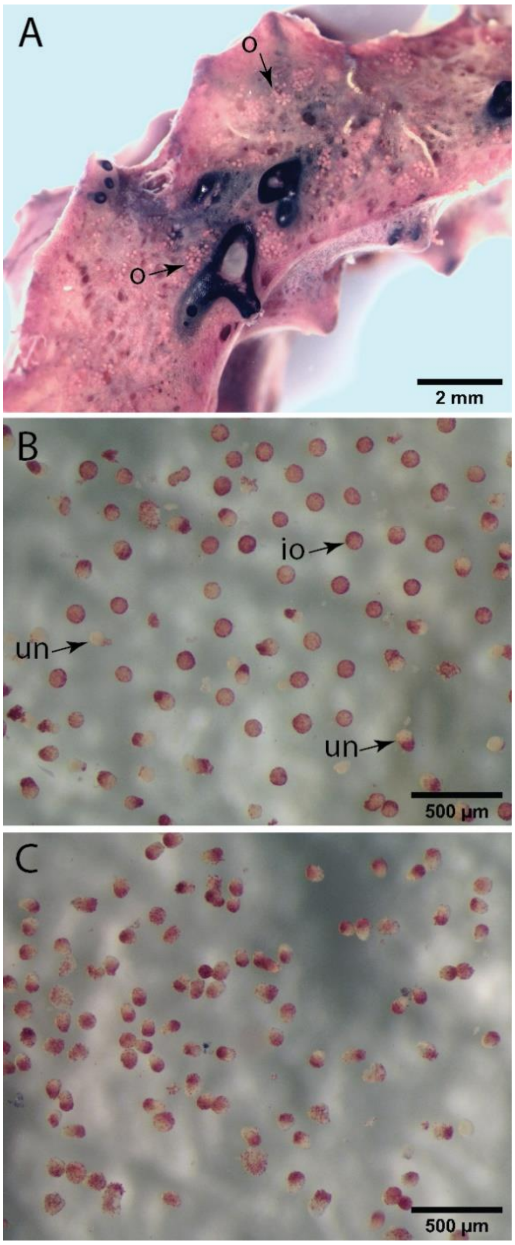

Figure S2. Macro- and micro-photographs of oocytes and larvae of *I. basta*. A) A cut through the sponge lamella showing the dense accumulation of oocytes in the adult mesohyl. B) Unfertilized oocytes; intact oocytes (io) appear as perfect spheres with the oocyte surrounded by a layer of maternal cells (dark red colouration). Damaged oocytes display the unsheathing (un) of the maternal cell layer from the oocyte (cream-coloured spheres). C) Developed larvae after 24h post-fertilization displaying elongation along the anterior (cream) – posterior (dark red) axis.

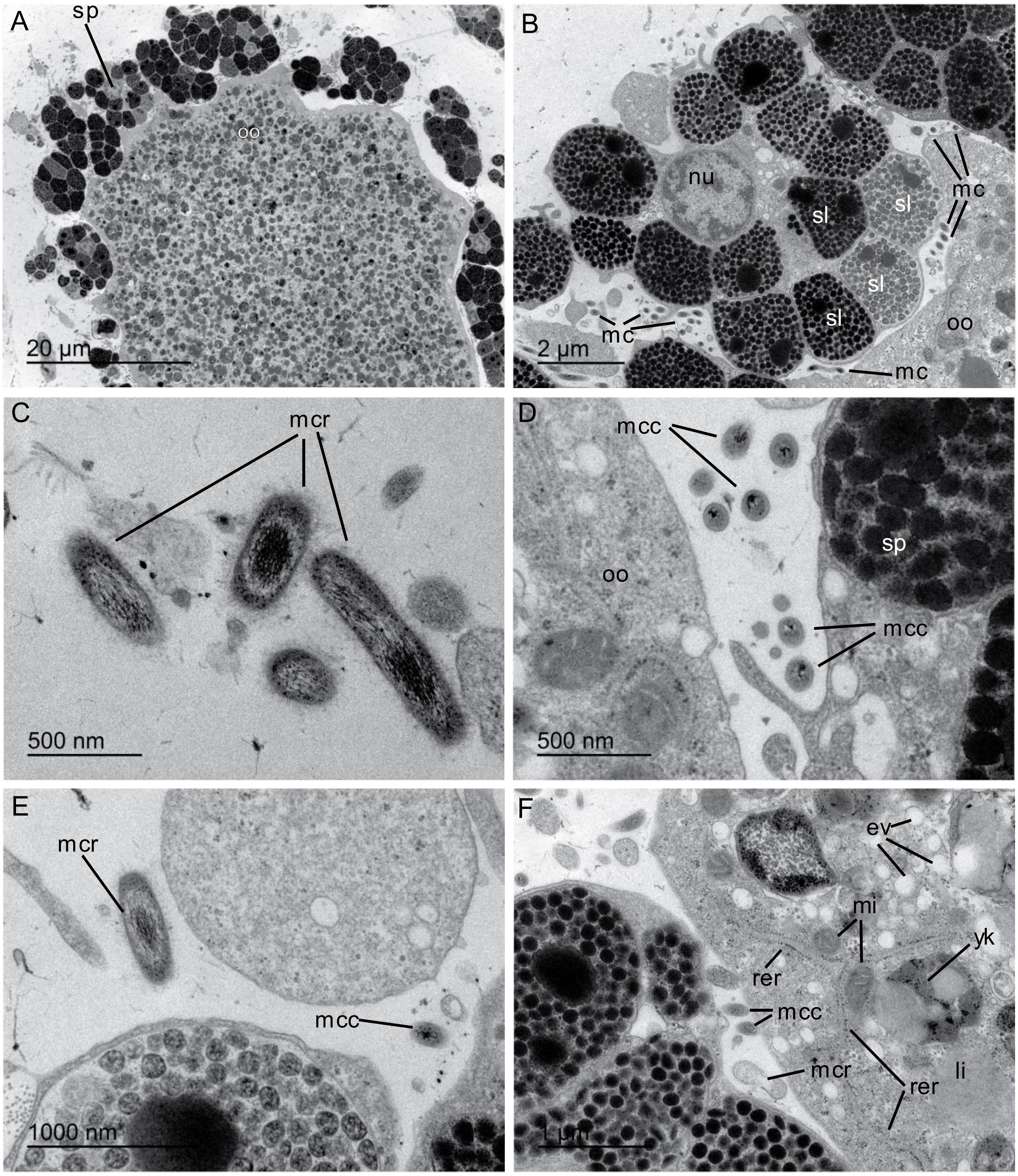

Figure S3. Transmission electron microscopy images of an unreleased egg in *Ianthella basta*. A) Partial view of a late-stage oocytes (oo) in female tissue sampled during the first day of spawning. Oocytes are surrounded by layer of maternal spherulous cells (sp). B) Detail of a maternal spherulous cell shown in panel A, which is embraced by the oocyte (oo) membrane. The nucleus (nu) of the cell is surrounded by spherules (sl), of which the granules are in different stages of maturation, as indicated by differences in their electron density. Microbes (mc) are present in the space between the spherulous cell and the

oocyte, named peri-oocytic space. C-E) Comparative views of microbes in the peri-oocytic space, which are either relatively large, rod-like microbial cells (mcr) or small, coccoid cells (mcc). F) Detailed view of the peripheral cytoplasm of an unreleased oocyte, showing rugose endoplasmic reticulum (rer), mitochondria (mi), electron clear vesicles (ev), yolk bodies (yk), and lipid droplets (li). Microbes (mcr, mcc) occur in the peri-oocytic space, but not within the cytoplasm of the oocyte.

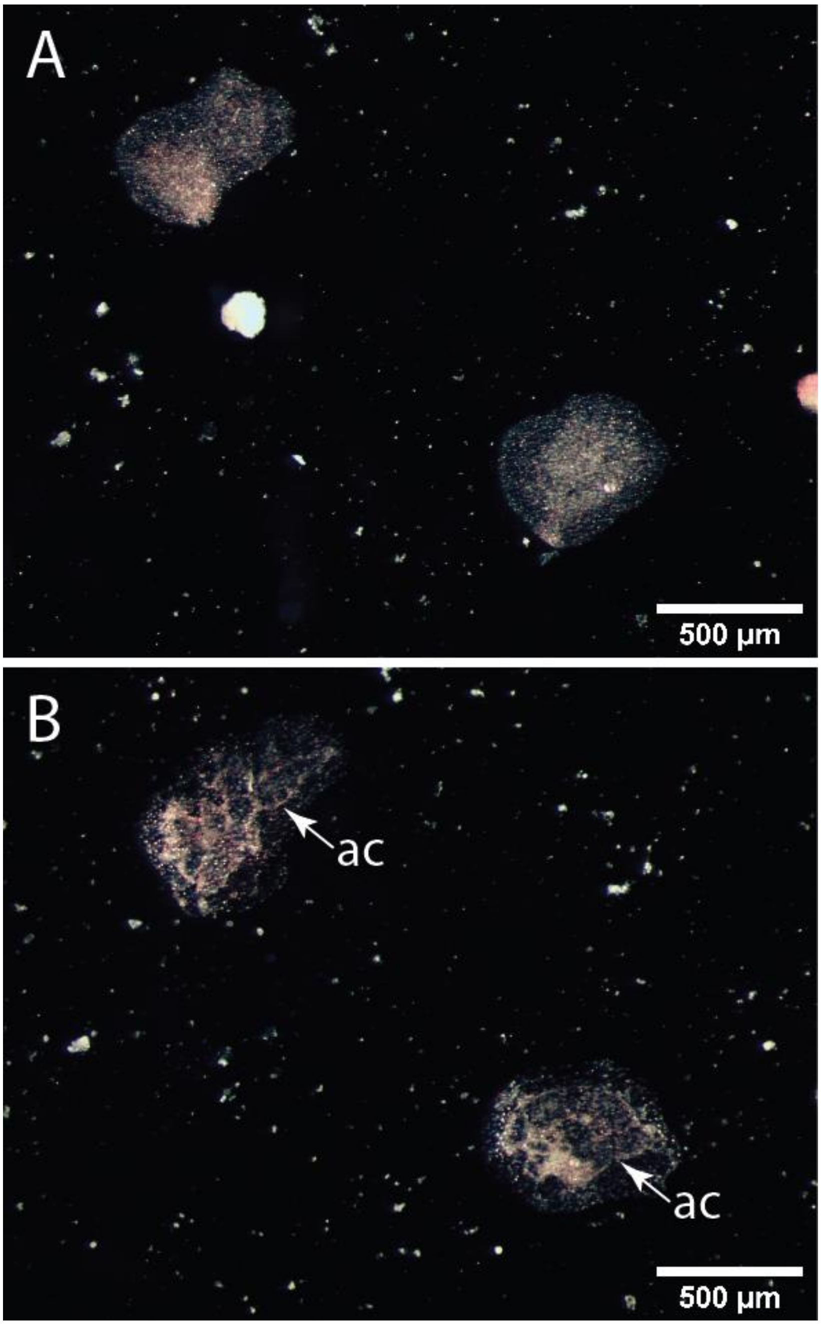

Figure S4. *I. basta* A) juveniles at 5-days post-settlement not having any visible aquiferous system development and B) the same juveniles at 12-days post-settlement displaying developed aquiferous canals (ac).

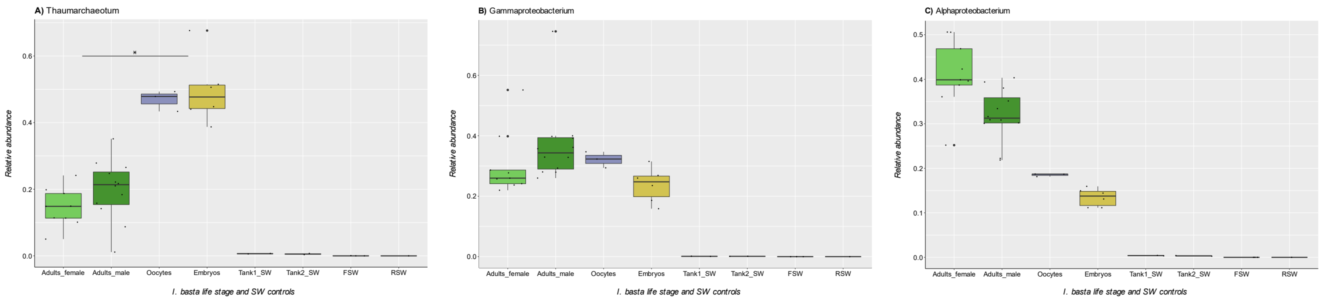

Figure S5. Relative abundance of the three dominant symbionts throughout *Ianthella basta*'s life stages and in the surrounding seawater. A) Thaumarchaeotum (*Candidatus* Nitrosospongia ianthellae, ASV_5060), B) Gammaproteobacterium (genus UBA10353_marine_group, ASV_803), and C) Alphaproteobacterium (unclassified, ASV_2141). The relative abundance of the Thaumarchaeotum was significantly different between adults and offspring (Aldex2; $p < 0.05$). Tank1_SW: Seawater in Tank 1, Tank2_SW: Seawater in Tank 2, FSW: Filtered seawater inflow, RSW: Raw seawater inflow.

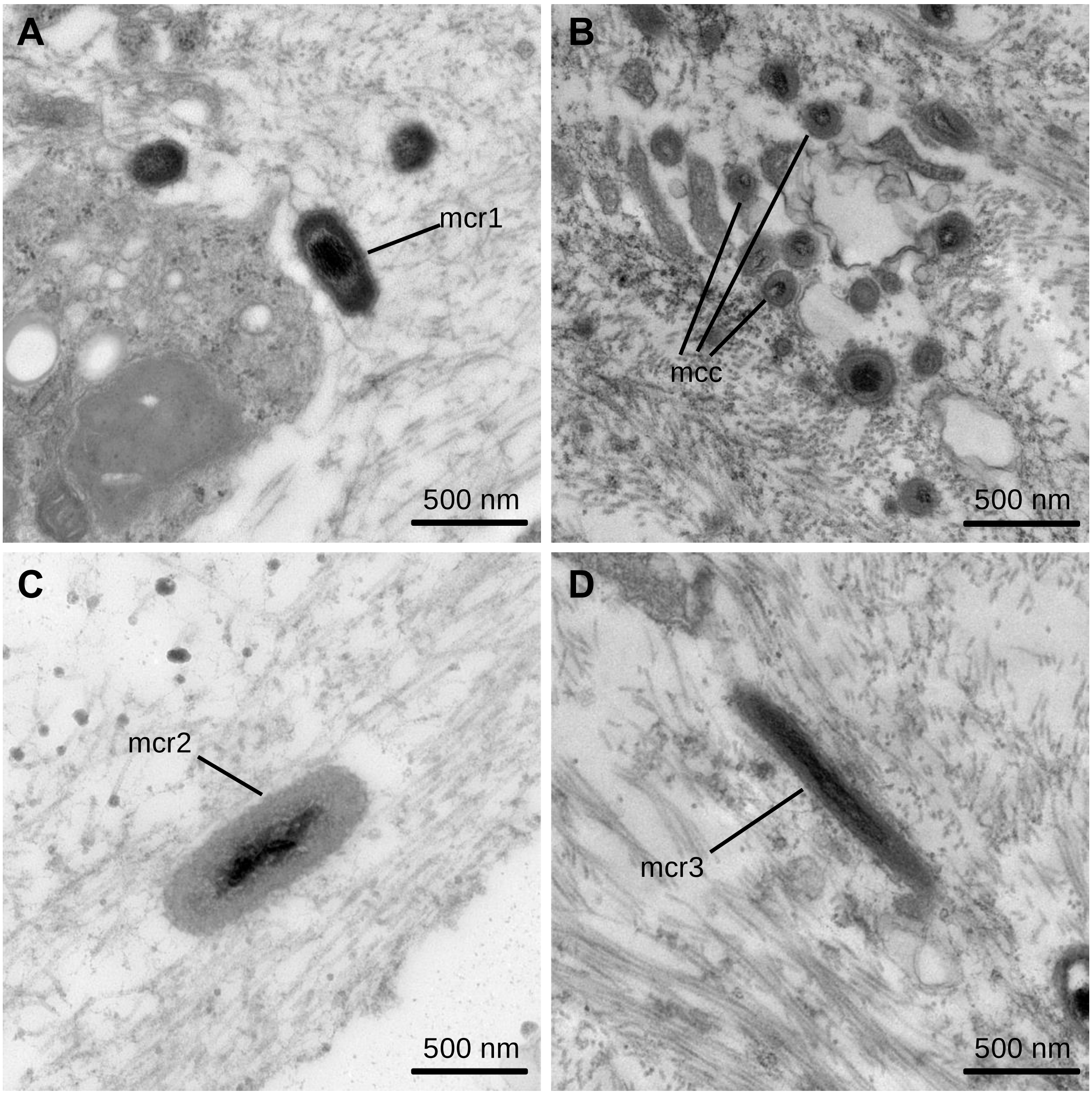

Figure S6. A-F) Dominant microbial morphotypes, i.e. rod-like (mcr) and cocci (mcc), of *I. basta* sampled in 2020 identified by using transmission electron microscopy (*n* = 3).

References

1. Schneider CA, Rasband WS, Eliceiri KW. NIH Image to ImageJ: 25 years of image analysis. Nat Methods. 2012;9(7):671-5.
